# Canine Leishmaniasis Control in the Context of One Health

**DOI:** 10.3201/eid2512.190164

**Published:** 2019-12

**Authors:** Filipe Dantas-Torres, Guadalupe Miró, Gad Baneth, Patrick Bourdeau, Edward Breitschwerdt, Gioia Capelli, Luís Cardoso, Michael J. Day, Gerhard Dobler, Luis Ferrer, Peter Irwin, Frans Jongejan, Volkhard A.J. Kempf, Barbara Kohn, Michael Lappin, Susan Little, Maxime Madder, Ricardo Maggi, Carla Maia, Mary Marcondes, Torsten Naucke, Gaetano Oliva, Maria Grazia Pennisi, Barend L. Penzhorn, Andrew Peregrine, Martin Pfeffer, Xavier Roura, Angel Sainz, SungShik Shin, Laia Solano-Gallego, Reinhard K. Straubinger, Séverine Tasker, Rebecca Traub, Ian Wright, Dwight D. Bowman, Luigi Gradoni, Domenico Otranto

**Affiliations:** Aggeu Magalhães Institute, Fundação Oswaldo Cruz (Fiocruz),; Recife, Brazil (F. Dantas-Torres);; Universidad Complutense de Madrid, Madrid, Spain (G. Miró, A. Sainz);; Hebrew University of Jerusalem, Rehovot, Israel (G. Baneth);; Ecole Nationale Vétérinaire, Nantes, France (P. Bourdeau);; North Carolina State University, Raleigh, North Carolina, USA (E. Breitschwerdt, R. Maggi);; Istituto Zooprofilattico Sperimentale delle Venezie, Legnaro, Italy (G. Capelli);; University of Trás-os-Montes e Alto Douro (UTAD), Vila Real, Portugal (L. Cardoso);; Murdoch University, Murdoch, Western Australia, Australia (M.J. Day, P. Irwin);; Bundeswehr Institute of Microbiology, Munich, Germany (G. Dobler);; Universitat Autònoma de Barcelona, Bellaterra, Spain (L. Ferrer, X. Roura, L. Solano-Gallego);; Utrecht University, Utrecht, the Netherlands (F. Jongejan);; Goethe-University, Frankfurt am Main, Germany (V.A.J. Kempf ); Freie Universität Berlin, Berlin, Germany (B. Kohn);; Colorado State University, Fort Collins, Colorado, USA (M. Lappin);; Oklahoma State University, Stillwater, Oklahoma, USA (S. Little);; Clinglobal, Tamarin, Mauritius (M. Madder); Universidade NOVA de Lisboa, Lisbon, Portugal (C. Maia);; São Paulo State University, São Paulo, Brazil (M. Marcondes); LABOKLIN GmbH, Bad Kissingen, Germany (T. Naucke);; University of Naples Federico II, Naples, Italy (G. Oliva); U; niversity of Messina, Messina, Italy (M.G. Pennisi); University of Pretoria, Onderstepoort, South Africa (B.L. Penzhorn);; University of Guelph, Guelph, Ontario, Canada (A. Peregrine);; Universität Leipzig, Leipzig, Germany (M. Pfeffer);; Chonnam National University, Gwangju, South Korea (S. Shin);; Ludwig-Maximilians-Universität München, Munich, Germany (R.K. Straubinger); University of Bristol, Bristol, UK (S. Tasker);; University of Melbourne, Parkville, Victoria, Australia (R. Traub);; The Mount Veterinary Practice, Fleetwood, UK (I. Wright); Cornell University, Ithaca, New York, USA (D.D. Bowman);; Istituto Superiore di Sanità, Rome, Italy (L. Gradoni); University of Bari Aldo Moro, Bari, Italy (D. Otranto)

**Keywords:** dogs, culling, control, zoonoses, pyrethroids, Leishmania, leishmaniasis, One Health, parasites, vector-borne infections

## Abstract

Dogs are the main reservoir of *Leishmania infantum* and in some countries have been regularly culled as part of government policy to control visceral leishmaniasis. At the 13th Symposium of the Companion Vector-Borne Diseases World Forum in Windsor, UK, March 19–22, 2018, we consolidated a consensus statement regarding the usefulness of dog culling as a means of controlling visceral leishmaniasis. The statement highlighted the futility of culling infected dogs, whether healthy or sick, as a measure to control the domestic reservoir of *L. infantum* and reduce the risk for visceral leishmaniasis.

Visceral leishmaniasis (VL), caused by *Leishmania donovani* in Asia and Africa and by *L. infantum* in the Mediterranean Basin, the Middle East, Central Asia, South America, and Central America, is a life-threatening disease that affects ≈200,000–400,000 persons annually and causes an estimated ≈20,000–40,000 deaths per year (*1*,*2*). Although an increasing number of other mammalian hosts, including infected humans, have served as effective reservoirs by infecting phlebotomine sand fly vectors, dogs remain a pivotal indirect source in many situations where the transmission cycle of *L. infantum* occurs (*3*,*4*).

Over the years, millions of dogs have been killed as part of government policies to control human VL caused by *L. infantum*, also known as zoonotic VL (*5*). The national public health policies of Central Asian, Caucasian, and some Balkan countries still recommend culling any *L. infantum*–positive dog (*1*). In rural areas of China, the Maghreb countries (North Africa), and parts of the Middle East, dog culling remains common practice (*1*), although medical therapy is usually allowed for dogs that are owned. In Central and South America, dog culling has been recommended and practiced in several countries, including Argentina, Brazil, Colombia, Uruguay, and Venezuela (*1*). Nonetheless, this practice has been replaced by more effective approaches, even in countries like Brazil, where thousands of dogs used to be culled every year (*5*).

The Companion Vector-Borne Diseases (CVBD) World Forum is a group of scientists working on canine and feline vectorborne diseases (*6*). This group contributes to an ongoing discussion and update on vectorborne diseases from around the world and their effects on dogs, cats, and humans. Because the topic of canine leishmaniasis is of global importance and thus frequently discussed, a consensus was reached that we should be more proactive in our position toward controlling this disease. At the 13th Symposium of the CVBD World Forum, held in Windsor, UK, during March 19–22, 2018, we discussed the control of canine leishmaniasis caused by *L. infantum* in the context of One Health and consolidated a consensus statement about the usefulness of dog culling as a means of controlling VL. This statement targets areas where VL caused by *L. infantum* is endemic and dog culling has been a common practice. We present this consensus statement and highlight the futility of culling infected dogs, whether clinically healthy or sick, as a measure to control the domestic reservoir of *L. infantum* and reduce the risk for VL in humans.

## Scientific Reasons Why Dog Culling Is Unacceptable

In areas of Asia (e.g., China) where government regimes have promoted massive culling of all dogs (regardless of seropositive status), in association with widespread use of DDT for vector control (*7*), the disease incidence declined for many years. However, whether this was an effect of dog elimination, vector control, or both is difficult to say (*5**,**7*). In fact, during the past 20 years, a mass of scientific evidence has accumulated from around the world and under different ecologic scenarios that demonstrates the failure of dog culling as a control strategy, particularly in Brazil (*5*,*7*). A dog culling strategy is not supportable for several reasons.

First, no reliable body of scientific evidence supports the effectiveness of dog culling as a means of reducing the incidence of VL (*8*,*9*). Second, alternative reservoir hosts may play a role in maintaining the life cycle of *L. infantum* (*3*,*4*) and must be taken into consideration when an integrated control strategy is formulated. Third, culled dogs are rapidly replaced with young dogs that are often more susceptible to primary infection (*10*). Fourth, serologic diagnostic tools often used for screening dogs as part of a culling program have limitations in terms of sensitivity and specificity (e.g., cross-reactivity where other *Leishmania* spp. or trypanosomatids occur) (*11*,*12*). Fifth, dog culling is not a cost-effective, valid alternative from a socioeconomic perspective (e.g., effect of dog removal on their owners and drugs for euthanasia) to government institutions (*7*), particularly in developing countries, that promises a long-term solution to the problem. Finally, effective control of *L. infantum* transmission requires integrated approaches focusing not only on the dog as an indirect source, but also the parasite and, importantly, the sand fly vector (*13*). Thus, the use of dog culling as a strategy to reduce the incidence of VL in humans cannot be justified and should no longer be used.

### Alternative and More Effective Solutions for Better Control of Canine Leishmaniasis

A plethora of scientific evidence demonstrates that the regular use of topical repellent insecticides is highly effective in preventing phlebotomine sand fly bites (*13*–*15*) and, therefore, *L. infantum* transmission (*16*,*17*). The constant use of repellent insecticides not only protects the dogs from sand flies infected on other hosts (and thus from becoming infected and acting as sources of infection) but also enables a reduction of these vectors in the vicinity of humans, potentially resulting in a reduction of human infections and clinical VL incidence (*18*,*19*). Vaccines (i.e., Leish-Tec, Ceva Saúde Animal Ltda, https://www.ceva.com.br/Produtos/Lista-de-Produtos/LEISH-TEC; CaniLeish, Virbac Schweiz AG, https://www.virbac.ch/de/kleintiere-produkte/impfstoffe/canileish; and LetiFend, Laboratorios LETI, Lda., https://saludanimal.leti.com/en/letifend-vaccine-against-canine-leishmaniasis_3944) are also available in some countries for reducing the risk for appearance of clinical signs and disease progression in infected dogs (*15*,*20*). Chemotherapy (e.g., allopurinol plus meglumine antimoniate or allopurinol plus miltefosine) and immune therapy (e.g., domperidone, and dietary nucleotides plus active hexose correlated compound) also may reduce the infectiousness of treated dogs, leading to a decrease of infected phlebotomine sand flies under experimental conditions (*15*,*21**–**25*).

### Our Consensus Advice and Practical Recommendations

All veterinarians take an oath, an example of which is: “I solemnly swear to use my scientific knowledge and skills for the benefit of society through the protection of animal health and welfare, the prevention and relief of animal suffering, the conservation of animal resources, the promotion of public health, and the advancement of medical knowledge” (https://www.avma.org/KB/Policies/Pages/veterinarians-oath.aspx). For the control of VL by *L. infantum*, scientific data clearly align closely with the sentiments expressed by all veterinarians in adhering to their oath, certain in the knowledge that preventive methods should be used, rather than the practice of dog culling, which we believe to be unethical and unjustifiable from a scientific viewpoint.

Using the basis of a One Health approach toward the prevention of zoonotic *Leishmania* infection in animals and humans, the members of the CVBD World Forum advocate the following recommendations concerning *L. infantum* infection in companion animals:

1. Companion animals should be protected from phlebotomine sand fly bites to prevent either leishmanial primary infection or spread from already infected dogs. Additional control measures, including environmental vector control, vaccination, and prophylactic medications (*14*,*15*,*26*), may also be used where available.

2. Dog culling in areas where VL is endemic should be replaced with alternative nonterminal measures that can prevent infection in dogs.

The members of the CVBD World Forum recommend the following measures to reduce the risk for *L. infantum* infection in dogs and in humans:

1. Promote phlebotomine sand fly bite prevention to reduce the risk for *L. infantum* infection in noninfected dogs and its spread from already infected dogs.

2. Improve the general health and nutritional status of dogs.

3. Implement latest concepts regarding the clinical management of canine leishmaniasis, including approaches to diagnosis and treatment.

4. Improve environmental and housing conditions to enhance phlebotomine sand fly control and reduce the exposure of humans to the vectors.
